# Supplementary material for: Discriminating protein tags on a dsDNA construct using a Dual Nanopore Device
Source: Sci Rep. 2022 Jul 4;12:11305. doi: 10.1038/s41598-022-14609-9 (PMC9253098; doi:10.1038/s41598-022-14609-9)
Supplement: Supplementary file 1 — Supplementary Information 1. [file 41598_2022_14609_MOESM1_ESM.pdf]

Supplementary Material for "Discriminating protein tags on a dsDNA construct using a Dual Nanopore Device"

Swarnadeep Seth[1], Arthur Rand[4], Walter Reisner[2], William B. Dunbar[4], Robert Sladek[3], and Aniket Bhattacharya[1]
[1] *Department of Physics, University of Central Florida, Orlando, Florida 32816-2385, USA*
[2] *Department of Physics, McGill University, 3600 rue university, Montreal, Quebec H3A 2T8, Canada*
[3] *Departments of Medicine & Human Genetics, McGill University, Montreal, H3A 0G1, Canada and*
[4] *Nooma Bio, 250 Natural Bridge Dr, Santa Cruz, CA 95060, USA*

I. BROWNIAN DYNAMICS (BD) SIMULATION DETAILS

We have coarse grained a 16.6μm long λ-phage dsDNA (please see the supplementary material-II) into 1024 beads with each bead having a diameter of σ. This translates to $\sigma \simeq 47$ bp $\simeq$ 16nm per bead. The polymer model was originally introduced for a fully flexible chain by Grest and Kremer [S1]. A short-ranged Lennard-Jones (LJ) potential

$$U_{\mathrm{LJ}}(r_{ij}) = 4\epsilon \left[\left(\frac{\sigma}{r_{ij}}\right)^{12} - \left(\frac{\sigma}{r_{ij}}\right)^{6} \right] + \epsilon, \text{ for } \ r_{ij} \le 2^{1/6}\sigma;$$
$$= 0, \text{ for } \ r_{ij} > 2^{1/6}\sigma. \tag{S1}$$

is used to model the excluded volume interaction of strength ϵ between the any two beads i and j separated by a distance r_{ij}. The connectivity between two neighboring beads is constructed using the finitely extensible nonlinear elastic (FENE) spring potential

$$U_{\mathrm{FENE}}(r_{ij}) = -\frac{1}{2}k_F R_0^2 \ln\left(1 - r_{ij}^2/R_0^2\right). \tag{S2}$$

Here, $j = i \pm 1$, so that $r_{ij} = |\vec{r}_i - \vec{r}_j|$ is the distance between two consecutive monomer beads, k_F is the spring constant, and R_0 is the maximum allowed separation between two connected monomers. An angle dependent three body interaction term is introduced between successive bonds which accounts for the chain stiffness κ

$$U_{\mathrm{bend}}(\theta_i) = \kappa\left(1 - \cos\theta_i\right) \tag{S3}$$

and θ_i is the angle between the bond vectors $\vec{b}_{i-1} = \vec{r}_i - \vec{r}_{i-1}$ and $\vec{b}_i = \vec{r}_{i+1} - \vec{r}_i$, respectively. For a homopolymer chain the persistence length ℓ_p in three dimensions (3D) is given by [S2]

$$\ell_p/\sigma = \kappa/k_BT. \tag{S4}$$

The double nanopore device is constructed by drilling two nanopores into a single thick slab and the nanopores are separated by a distance of 32σ which translates to 550 nm pore-to-pore separation in the experiment. The left nanopore of diameter 7σ and the right nanopore of diameter 6σ are constructed by removing out particles from the 6σ thick top slab consists of immobile LJ particles. Two reservoirs having a depth and width of 96σ are attached below the left and right pores respectively (shown in the Fig.1 in the main article). We use the Brownian dynamics simulation following the equations of motion for the ith monomer

$$m_i\ddot{\vec{r}}_i = -\nabla(U_{\mathrm{LJ}} + U_{\mathrm{FENE}} + U_{\mathrm{bend}} + U_{\mathrm{wall}}) - \Gamma_i\vec{v}_i + \vec{\eta}_i. \tag{S5}$$

Here $\vec{\eta}_i(t)$ is the Gaussian white noise with zero mean at temperature T, and satisfies the fluctuation-dissipation relation in d physical dimensions (here $d = 3$):

$$< \vec{\eta}_i(t) \cdot \vec{\eta}_j(t') >= 2dk_BT\Gamma_i\,\delta_{ij}\,\delta(t - t'). \tag{S6}$$

We express length and energy in units of σ and ϵ, respectively. The parameters for the FENE potential in Eq. (S2), k_F and R_0, are set to $k_F = 30\epsilon/\sigma$ and $R_0 = 1.5\sigma$, respectively. The friction coefficient and the temperature are set to $\Gamma_i = 0.7\sqrt{m_i\epsilon/\sigma^2}$ and $k_BT/\epsilon = 1.0$. The numerical integration of Equation (S5) is implemented using the algorithm introduced by Gunsteren and Berendsen [S3]. Our previous experiences with BD simulation suggests that for a time step $\Delta t = 0.01$ these parameters values produce stable trajectories over a very long period of time and do not lead to unphysical crossing of a bond by a monomer [S4, S5]. The average bond length stabilizes to $\langle b_l\rangle = 0.971 \pm 0.001\sigma$ with negligible fluctuation regardless of the chain size and rigidity [S4]. Hence, we relate the polymer's contour length L and the number of monomers N as $L = (N-1)\langle b_l\rangle$.

II. SIMULATION MOVIE

The simulation movie (generated using the VMD software [S6]) will help to understand further how the capture and flossing of a dsDNA works in a double nanopore system.

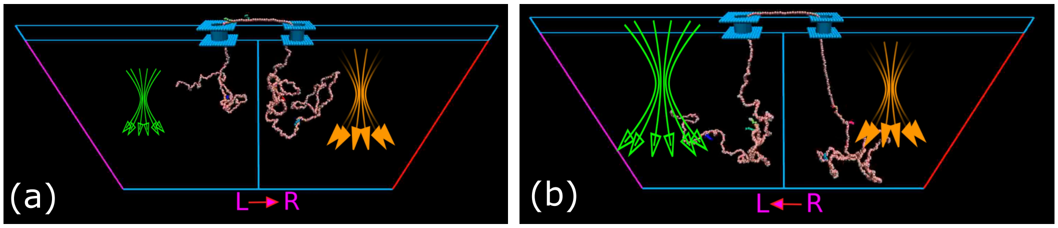

FIG. S1. (a) A single movie frame (screen-shot) depicting the $L \rightarrow R$ scan the dsDNA through the double nanopore setup in presence of the extended electric fields. (b) The frame shows the dsDNA during the $R \rightarrow L$ scan. In both cases, the left/right nanopre forces are shown in green/orange arrows (not to scale). The dsDNA backbone monomers are depicted using the pink beads and the seven sidechain tags are shown in their respective colors.

[S1] Grest, G. S.; Kremer, K. Molecular Dynamics Simulation for Polymers in the Presence of a Heat Bath. Phys. Rev. A, **33** (5), 3628-3631 (1986).

[S2] Landau, L. D.; Lifshitz, E. M.Statistical Physics; Pergamon Press (1981).

[S3] van Gunsteren, W. F., & Berendsen, H. J. C. Algorithms for brownian dynamics. Molecular Physics, **45**, 637-647 (1982).

[S4] Huang, A.; Bhattacharya, A.; Binder, K. Conformations, Transverse Fluctuations, and Crossover Dynamics of a Semi-Flexible Chain in Two Dimensions. The Journal of Chemical Physics **140** (21), 214902 (2014).

[S5] Huang, A.; Adhikari, R.; Bhattacharya, A.; Binder, K. Universal Monomer Dynamics of a Two-Dimensional Semi-Flexible Chain. EPL **105** (1), 18002 (2014).

[S6] Humphrey, W., Dalke, A. & Schulten, K., "VMD: Visual Molecular Dynamics" J. Molec. Graphics, **14**, 33-38 (1996).
