# Supplementary material for: Discriminating protein tags on a dsDNA construct using a Dual Nanopore Device
Source: Sci Rep. 2022 Jul 4;12:11305. doi: 10.1038/s41598-022-14609-9 (PMC9253098; doi:10.1038/s41598-022-14609-9)
Supplement: Supplementary file 2 — Supplementary Information 2. [file 41598_2022_14609_MOESM2_ESM.pdf]

Supplementary Material for "Discriminating protein tags on a dsDNA construct using a Dual Nanopore Device"

Swarnadeep Seth[1], Arthur Rand[4], Walter Reisner[2], William B. Dunbar[4], Robert Sladek[3], and Aniket Bhattacharya[1]
[1]*Department of Physics, University of Central Florida, Orlando, Florida 32816-2385, USA*
[2]*Department of Physics, McGill University, 3600 rue university, Montreal, Quebec H3A 2T8, Canada*
[3]*Departments of Medicine & Human Genetics, McGill University, Montreal, H3A 0G1, Canada and*
[4]*Nooma Bio, 250 Natural Bridge Dr, Santa Cruz, CA 95060, USA*

II. EXPERIMENTAL PROTOCOL

Experimental results for tag dwell-times in our dual-pore device are obtained using previously published methods. Device fabrication is described in Zhang *et al.* [S1], use of FPGA protocol for efficiently forming tug-of-war states is described in Li *et al.* 2019 [S2], basic multi-scanning protocols and tag blockade analysis are described in Li *et al.* 2020 [S3] and zoom multi-scanning and recapture protocols are described in Rand *et al.* [S4].

Briefly, the labeled DNA constructs used are based on λ-DNA with 90 nt long oligo-flap labels inserted at the recognition sites of the nicking enzyme Nt.BbvC1. The labels are generated by using nick-translation to insert azide bearing nucleotides at the Nt.BbvC1 recognition sites; these are then coupled to 90 nt long DBCO bearing moieties via copper-free click chemistry [S4]. The labeled constructs are introduced into a dual-pore device in 2 M LiCl buffer containing two 20-30 nm diameter pores fabricated via focused ion-beam (FIB) [S1]. The pores have a spacing of around 500 nm. Each pore is coupled to a separate microchannel that can be independently biased with respect to a common reservoir giving access to both pores. The current through each pore is measured using a dual channel recorder (Molecular Device Multi-clam 700B) interfaced to a Field-Programmable Gate Array (FPGA, PCIe-7851R). The FPGA can apply active logic to exploit feedback signals, in the form of measured currents at each pore, to dynamically adjust the dual pore biasing during a translocation event. The signals are sampled at 250 kHz and filtered at 10 kHz. Specifically, the results obtained correspond to the following parameters: pore 1 has a diameter of 23.9 nm, pore 2 has a diameter of 20.4 nm and the pore-to-pore spacing is 561 nm (obtained from SEM).

The labeled λ-DNA is captured in a tug-of-war state [S2] and then subjected to a multi-scanning protocol where the molecule is driven back and forth between the pores ([S3, S4], which we term "flossing"). In a dual-pore device, when a molecule trapped in a tug-of-war state, the translocation speed and polarity is determined fundamentally by the magnitude and sign of the biasing differential between the pores. In our scanning protocol, to achieve either L-R or R-L directed motion, the voltage at one pore, which we will term pore 2, is held fixed (at 300 mV) and the voltage at pore 1 is varied between a low (150 mV, for L-R motion) and high level (650 mV, for R-L motion) with respect to the pore 1 voltage. In order to vary the scan polarity in a systematic way to achieve multi-scanning, the FPGA is then programmed to count the number of tags that pass through a pore (say pore 2 for a translocation in the L-R direction), and then reverse the translocation polarity from the L-R direction to the R-L direction when a fixed number of tags have passed through the pore. The number of tags that pass through pore 1 is then counted and the translocation polarity flipped when a fixed number have passed through. Repetition of these counting cycles at pore 1 and 2 leads to repetitive back-and-forth cycling of the chain. In the zoom multi-scanning protocol [S4], the number of tags that are allowed to pass through pore 1 and 2 is gradually increased, leading to scanning of successively larger portions of the chain. In addition, the FPGA can recapture the same chain to perform multiple multi-scanning cycles on the same chain [S4]. The tag results shown correspond to 18 separate λ-DNA molecules captured and 46 recapture events. The translocation velocity v can be measured by finding the time-of-flight (TOF) for a tag to move between the pores: $v = \langle TOF \rangle / d$ where d is the pore-to-pore spacing and $\langle TOF \rangle$ is the average TOF over all tags measured for a given captured molecule (including all individual recaptures for the given molecule). Averaging the translocation velocity further over all molecules captured gives: $v_{L-R} = 0.99 \pm 0.01$ (for L-R direction) and $v_{R-L} = 2.23 \pm 0.05$ (for R-L direction). Error given corresponds to error on the mean over all 18 molecules captured.

The tag-blockades are analyzed on individual scans using the approach developed in [S3] coded in Matlab (see in particular supplementary methods for this article). First, the positions of the tags along the scan are identified using a peak-finding algorithm. Next, we model the varying current background arising from capacitance transients introduced by flipping the voltage. To do this, a fixed region of around 400 μs is removed about the position of each identified tag and then the remaining signal, giving purely the varying current background, is fit to a spline. The spline model for the varying background is then substracted from the signals for pore 1 and pore 2. The individual

blockade profiles for each tag are then modeled via the fitting function:

$$I(t) = I_{b1} + I_{b2}t - \frac{I_o}{2}\left[\text{erf}\left(\frac{t-(t_o-\Delta t/2)}{\sqrt{2}\sigma}\right) - \text{erf}\left(\frac{t-(t_o+\Delta t/2)}{\sqrt{2}\sigma}\right)\right] \tag{S1}$$

This form is based on convolving a box of width Δt, height I_o centered at position t_o with a Gaussian of width σ. In the limit that $\Delta t \gg \sigma$, the model yields a box-like profile. In the limit that $\Delta t \ll \sigma$, the model yields a Gaussian-like profile. The profile width at half-maximum is reported as the pore dwell-time (note that the width at half-maximum is obtained numerically as a function of Δt and σ from the blockade model). The parameters I_{b1}, I_{b2}, which specify a constant and linear term, help characterize residual varying background at the tag-position. The 46 recapture events obtained from the 18 molecules captured corresponds with multi-scanning to 2256 measured dwell-time pairs (e.g. for pore 1 and pore 2).

[S1] Zhang, Y., Liu, X., Zhao, Y., Yu, J.-K., Reisner, W. & Dunbar, W. B. Single Molecule DNA Resensing Using a Two-Pore Device. Small **14**, 1801890 (2018).

[S2] Liu, X., Zhang, Y., Nagel, R., Reisner, W. & Dunbar, W. B. Controlling DNA Tug-of-War in a Dual Nanopore Device. Small **15**, 1901704 (2019).

[S3] Liu, X., Zimny, P., Zhang, Y., Rana, A., Nagel, R., Reisner, W. & Dunbar, W. B. Flossing DNA in a Dual Nanopore Device. Small **16**, 1905379 (2020).

[S4] Rand, A., Zimny, P., Nagel, R., Telang, C., Mollison, J., Bruns, A., Leff, E., Reisner, W. W., & Dunbar, W. B. Electronic Mapping of a Bacterial Genome with Dual Solid-State Nanopores and Active Single-Molecule Control. ACS Nano, **16**, 5258-5273 (2022).
