# Supplementary material for: Discriminating protein tags on a dsDNA construct using a Dual Nanopore Device
Source: Sci Rep. 2022 Jul 4;12:11305. doi: 10.1038/s41598-022-14609-9 (PMC9253098; doi:10.1038/s41598-022-14609-9)
Supplement: Supplementary file 3 — Supplementary Information 3. [file 41598_2022_14609_MOESM3_ESM.pdf]

Supplementary Material for "Discriminating protein tags on a dsDNA construct using a Dual Nanopore Device"

Swarnadeep Seth[1], Arthur Rand[4], Walter Reisner[2], William B. Dunbar[4], Robert Sladek[3], and Aniket Bhattacharya[1]
[1]*Department of Physics, University of Central Florida, Orlando, Florida 32816-2385, USA*
[2]*Department of Physics, McGill University, 3600 rue university, Montreal, Quebec H3A 2T8, Canada*
[3]*Departments of Medicine & Human Genetics, McGill University, Montreal, H3A 0G1, Canada and*
[4]*Nooma Bio, 250 Natural Bridge Dr, Santa Cruz, CA 95060, USA*

III. NANOPORE ASYMMETRY

In the recent double nanopore experiments [S1–S4] there is a slight asymmetry in the pore diameters (please see the Supplementary Materials II). To compare the effect of the nanopore asymmetry on the cumulative dwell time distributions, we simulated two differnt systems (i) with symmetric pores (Fig. S1 ((a)-(b))) of diameter 6σ each and (ii) with a 16% asymmetric pores (Fig. S1((c)-(d))) where the left pore diameter is 7σ, and the right pore diameter is 6σ. We observe that a 16% asymmetry in the pore diameters does not affect the dwell time distributions qualitatively.

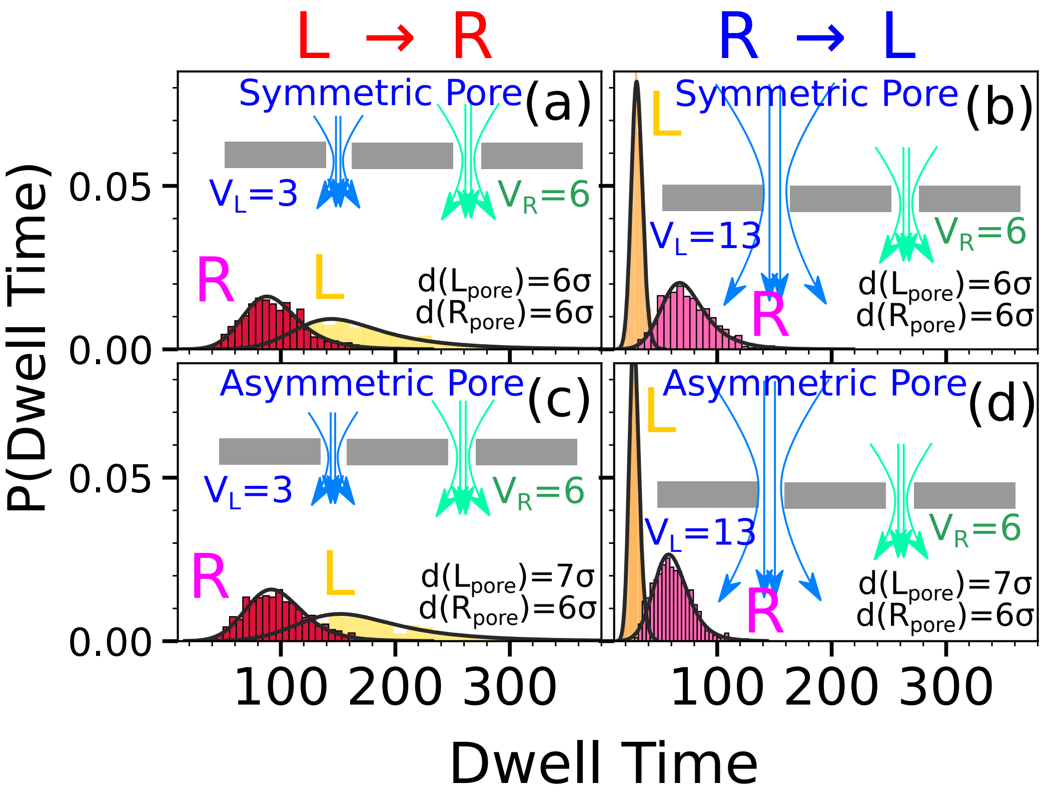

FIG. S1. Cumulative dwell time distributions of the seven tags (sidechains) (a) $L \to R$ and (b) $R \to L$ (1st row) for the equal left and right nanopore diameter of 6σ. The dwell time distributions for the left pore diameter 7σ and right pore diameter 6σ are shown for (c) $L \to R$ and (d) $R \to L$ scans (2nd row). In each row the yellow/red (left column) and the orange/magenta (right column) dwell time histograms are obtained from the left/right pore in $L \to R$ and $R \to L$ directions. For the $L \to R$ (left column) scans the left/right pore voltages are $V_L = 3$ and $V_R = 6$ respectively. For the $R \to L$ (right column) scans the left pore voltage is changed to $V_L = 13$ while right pore voltage remains unaltered. Schematics of the electrostatic forces on the DNA in left/right pore are shown by the blue/green arrows (not to scale). The black envelops represent the exponentially modified Gaussian distribution fit of the dwell time histograms.

[S1] Zhang, Y., Liu, X., Zhao, Y., Yu, J.-K., Reisner, W. & Dunbar, W. B. Single Molecule DNA Resensing Using a Two-Pore Device. Small **14**, 1801890 (2018).

[S2] Liu, X., Zhang, Y., Nagel, R., Reisner, W. & Dunbar, W. B. Controlling DNA Tug-of-War in a Dual Nanopore Device. Small **15**, 1901704 (2019).

[S3] Liu, X., Zimny, P., Zhang, Y., Rana, A., Nagel, R., Reisner, W. & Dunbar, W. B. Flossing DNA in a Dual Nanopore Device. Small **16**, 1905379 (2020).

[S4] Rand, A., Zimny, P., Nagel, R., Telang, C., Mollison, J., Bruns, A., Leff, E., Reisner, W. W., & Dunbar, W. B. Electronic Mapping of a Bacterial Genome with Dual Solid-State Nanopores and Active Single-Molecule Control. ACS Nano, **16**, 5258-5273 (2022).
