# Supplementary material for: Discriminating protein tags on a dsDNA construct using a Dual Nanopore Device
Source: Sci Rep. 2022 Jul 4;12:11305. doi: 10.1038/s41598-022-14609-9 (PMC9253098; doi:10.1038/s41598-022-14609-9)
Supplement: Supplementary file 4 — Supplementary Information 4. [file 41598_2022_14609_MOESM4_ESM.pdf]

Supplementary Material for "Discriminating protein tags on a dsDNA construct using a Dual Nanopore Device"

Swarnadeep Seth[1], Arthur Rand[4], Walter Reisner[2], William B. Dunbar[4], Robert Sladek[3], and Aniket Bhattacharya[1]
[1]*Department of Physics, University of Central Florida, Orlando, Florida 32816-2385, USA*
[2]*Department of Physics, McGill University, 3600 rue university, Montreal, Quebec H3A 2T8, Canada*
[3]*Departments of Medicine & Human Genetics, McGill University, Montreal, H3A 0G1, Canada and*
[4]*Nooma Bio, 250 Natural Bridge Dr, Santa Cruz, CA 95060, USA*

IV. EXTENDED ELECTRIC FIELD IN THE SIMULATION

We study the case where the electric field extends beyond the nanopore region in both the pores (please see the Supplementary Materials-I). We use the Finite Element method to solve the Poisson equation to construct the electric field around the nanopores using the FEniCS package [S1, S2]. In the left nanopore cavity, front, back, and the left side (away from the nanopore) are kept at a potential V_L. A similar voltage boundary condition also applies in the right nanopore where front, back, and the right side (away from the pore) are kept at a constant potential V_R. The other sides of the cavities are grounded at zero potential. The left pore voltage (V_L) is varied to multi-scan the DNA as discussed in the main manuscript. We obtain the potential $V(x,y,z)$ in the nanopore as well as in the outside volume by solving the Poisson equation at spacial three dimensional (3d) grid points of spacing $a = \sigma$ (σ is the diameter of a coarse-grained bead). While solving the boundary value problem, the Dirichlet boundary condition is applied on the sides of finite potentials and otherwise, Neumann boundary condition is maintained. The electric field $E(x,y,z)$ is obtained by taking the negative gradient of the potential $V(x,y,z)$ in the 3d spacial dimension. We implement the electric fields in each of the nanopore and the attched reservior seperately as the electric field fades rather fast away from the nanopore regions (please see the electric field in Fig. S1). Each of the DNA coarse-grained bead contains a unity amount of charge $|q| = 1.0$ and the spherical/sidechains tags are partially charged (Please refer to the main article for the details of the tag's partial charge). In presence of this spatially extended electric field each bead (with an index i) experiences an additional force $\vec{F}_{electric}(i) = q_i\vec{E}(x,y,z)$ depending upon the position of the bead in the double nanopore setup. An example of the electric field for a bias voltage $V = 10$ is shown in Fig. S1.

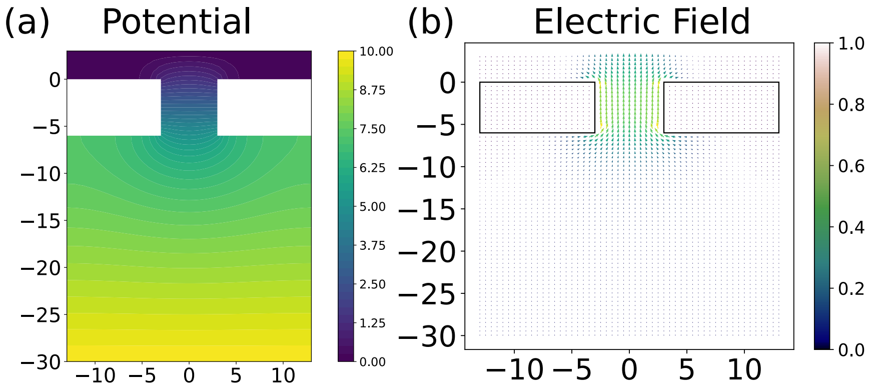

FIG. S1. (a) The figure shows the two diemensional (2d) projection of the potential in the visinity of a 6σ thick nanopore for an applied bias $V = 10$. Please note that we show the zoomed view of a single nanopore instead of the both pores for a better visulization. (b) The corresponding electric field is calulated by taking the negative gradinet of the potential. The side colorbars represent the scales for potential and electric field respectively.

[S1] Logg, A. & Wells, G. N., DOLFIN: Automated Finite Element Computing, ACM Transactions on Mathematical Software **37** (2010). doi:10.1145/1731022.1731030

[S2] Logg, A., Mardal, K.-A., & Wells, G. (Eds.). Automated Solution of Differential Equations by the Finite Element Method. Lecture Notes in Computational Science and Engineering, Springer (2012). doi:10.1007/978-3-642-23099-8
