# Supplementary material for: Discriminating protein tags on a dsDNA construct using a Dual Nanopore Device
Source: Sci Rep. 2022 Jul 4;12:11305. doi: 10.1038/s41598-022-14609-9 (PMC9253098; doi:10.1038/s41598-022-14609-9)
Supplement: Supplementary file 5 — Supplementary Information 5. [file 41598_2022_14609_MOESM5_ESM.pdf]

Supplementary Material for "Discriminating protein tags on a dsDNA construct using a Dual Nanopore Device"

Swarnadeep Seth[1], Arthur Rand[4], Walter Reisner[2], William B. Dunbar[4], Robert Sladek[3], and Aniket Bhattacharya[1]
[1]*Department of Physics, University of Central Florida, Orlando, Florida 32816-2385, USA*
[2]*Department of Physics, McGill University, 3600 rue university, Montreal, Quebec H3A 2T8, Canada*
[3]*Departments of Medicine & Human Genetics, McGill University, Montreal, H3A 0G1, Canada and*
[4]*Nooma Bio, 250 Natural Bridge Dr, Santa Cruz, CA 95060, USA*

V. SPHERICAL TAGS AND SIDECHAINS COMPARISON

We compare the cumulative dwell time distributions of the seven spherical tags and the sidechain tags. The spherical tag is heavier in mass ($6m$) and carries a charge $3q$. On contrary, the sidechains are of length $l_{tag} = 6\sigma$ with each of the sidechain beads is of mass m and carries a charge $0.5q$. Fig. S1 ((a)/(b)) show the dwell time distributions of the $L \to R/R \to L$ scans for the spherical tags and Fig. S1 ((c)/(d)) shows the same for the sidechains. We observe that both the spherical and sidechain tags produce asymmetry in the dwell time. For the $R \to L$ scans the dwell time asymmetry is more prominent for the sidechains which matches closer to the experiment.

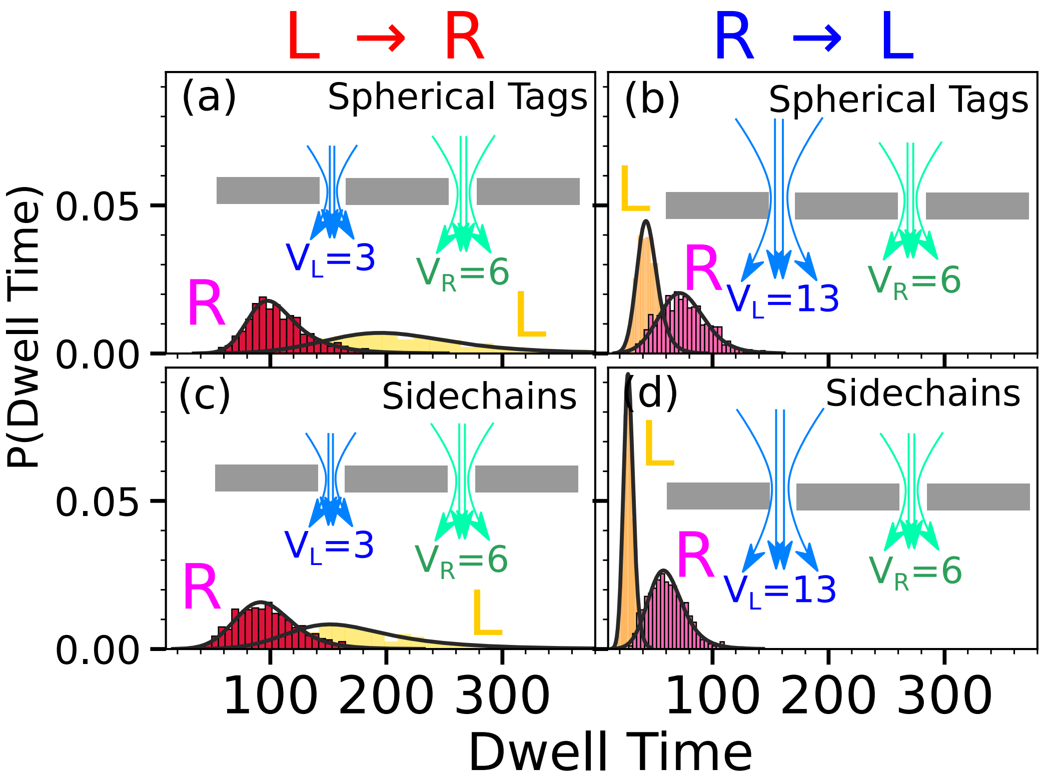

FIG. S1. Cumulative dwell time distributions (a) $L \to R$ and (b) $R \to L$ scans for the seven charged ($3q$) spherical tags with heavier mass ($6m$). The next row represent the cumulative dwell time distributions for (c) $L \to R$ and (d) $R \to L$ scans for the charged ($q_{tag} = 0.5q$) sidechain tags of length ($l_{tag} = 6\sigma$) For the both cases, for $L \to R$ translocations left pore voltage is $V_L = 3$ and right pore voltage is $V_R = 6$ while for the $R \to L$ translocations $V_L = 13$ and $V_R = 6$. The yellow/red and the orange/magenta dwell time histograms are obtained from the left/right pore in $L \to R$ and $R \to L$ directions respectively. Schematics of the electrostatic forces on the DNA in the left/right pore are shown by the blue/green arrows (not to scale). The black envelops represent the exponentially modified Gaussian distribution fit of the dwell time histograms.
