# Supplementary material for: Discriminating protein tags on a dsDNA construct using a Dual Nanopore Device
Source: Sci Rep. 2022 Jul 4;12:11305. doi: 10.1038/s41598-022-14609-9 (PMC9253098; doi:10.1038/s41598-022-14609-9)
Supplement: Supplementary file 6 — Supplementary Information 6. [file 41598_2022_14609_MOESM6_ESM.pdf]

Supplementary Material for "Discriminating protein tags on a dsDNA construct using a Dual Nanopore Device"

Swarnadeep Seth[1], Arthur Rand[4], Walter Reisner[2], William B. Dunbar[4], Robert Sladek[3], and Aniket Bhattacharya[1]

[1]*Department of Physics, University of Central Florida, Orlando, Florida 32816-2385, USA*

[2]*Department of Physics, McGill University, 3600 rue university, Montreal, Quebec H3A 2T8, Canada*

[3]*Departments of Medicine & Human Genetics, McGill University, Montreal, H3A 0G1, Canada and*

[4]*Nooma Bio, 250 Natural Bridge Dr, Santa Cruz, CA 95060, USA*

VI. INDIVIDUAL DWELL TIME DISTRIBUTION OF THE TAGS FROM THE SIMULATION

In the Brownian dynamics simulation the dwell time distributions of the partially charged sidechain tags are calculated from the residence time in the left/right nanopore regions for $L \rightarrow R/R \rightarrow L$ scans shown in the Fig. S1 (a) and (b) respectively. During the $L \rightarrow R$ scans for an individual tag, the left pore dwell time distribution is wider than the right pore and the opposite is observed for the $R \rightarrow L$ scans. It is also worth mentioning that for the closely separated tag pairs (T_2, T_3 and T_4, T_5, T_6) the dwell time increases the most for the left pore during the $L \rightarrow R$ scans and in the right pore during the $R \rightarrow L$ scans.

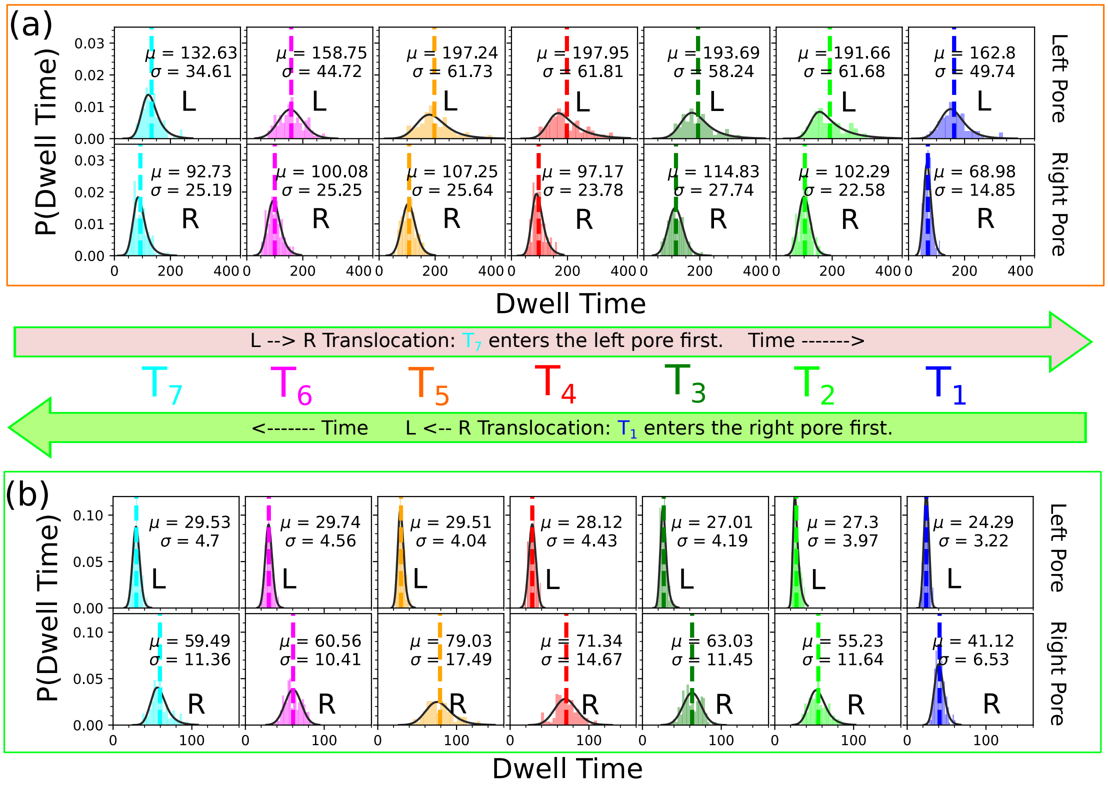

FIG. S1. The panel figures show the individual dwell time distribution of the seven sidechain tags obtained from the BD simulation for the voltages $V_L = 3$ and $V_R = 6$ for the $L \rightarrow R$ scans, and for $R \rightarrow L$ scans the left/right pore voltages are $V_L = 13$ and $V_R = 6$ respectively. (a) The first/second row represents the dwell time of the tags in the left/right pore respectively during the $L \rightarrow R$ scans. (b) The dwell time distributions for the individual sidechain tags for the $R \rightarrow L$ scans. The black dotted lines represent dwell time averages with the average values mentioned in the μ. The standard deviations are denoted by the σ. The black envelops represent the exponentially modified Gaussian distribution fit of the dwell time histograms.
