# Supplementary material for: Discriminating protein tags on a dsDNA construct using a Dual Nanopore Device
Source: Sci Rep. 2022 Jul 4;12:11305. doi: 10.1038/s41598-022-14609-9 (PMC9253098; doi:10.1038/s41598-022-14609-9)
Supplement: Supplementary file 7 — Supplementary Information 7. [file 41598_2022_14609_MOESM7_ESM.pdf]

Supplementary Material for "Discriminating protein tags on a dsDNA construct using a Dual Nanopore Device"

Swarnadeep Seth[1], Arthur Rand[4], Walter Reisner[2], William B. Dunbar[4], Robert Sladek[3], and Aniket Bhattacharya[1]

[1]*Department of Physics, University of Central Florida, Orlando, Florida 32816-2385, USA*

[2]*Department of Physics, McGill University, 3600 rue university, Montreal, Quebec H3A 2T8, Canada*

[3]*Departments of Medicine & Human Genetics, McGill University, Montreal, H3A 0G1, Canada and*

[4]*Nooma Bio, 250 Natural Bridge Dr, Santa Cruz, CA 95060, USA*

VII. COMPARISON OF EXPERIMENT AND SIMULATION DWELL TIME DISTRIBUTIONS FOR DIFFERENT VOLTAGE BIASES

In the double nanopore experiments [S1–S4] there is an uncertainty in the applied voltage on the left nanopore for the $R \to L$ scans. For the $L \to R$ scans the left pore voltage is $V_L = 150$ mV and the right pore voltage is 300 mV, and for the $R \to L$ scan the left pore voltage is increased to 600/650 mV while keeping the right pore applied voltage constant at 300 mV. In our Brownian dynamics simulation we have implemented both the voltage conditions, where $V_L/V_R = 0.5$ for the $L \to R$ scans and $V_L/V_R = 2.0$ shown in Fig. S1(d), and $V_L/V_R = 2.17$ shown in Fig. S1(f) for the $R \to L$ scans. We observe that this voltage variations in the left pore for $R \to L$ scans does not affect the distributions qualitatively.

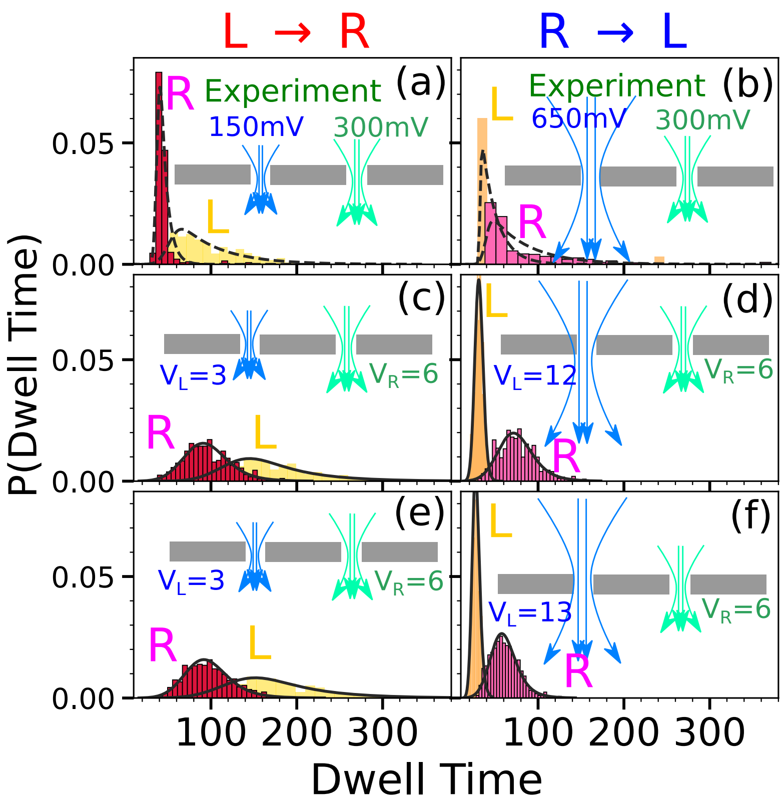

FIG. S1. Cumulative dwell time distributions of the seven tags (sidechains) (a) $L \to R$ and (b) $R \to L$ (1st row) obtained from the experiment. The next two rows (2nd - 3rd) are simulation dwell time data for different combinations of voltages V_L and V_R applied across the left and right nanopore. In the 2nd row, the ratio of the left/right pore voltages for (c) $L \to R$ scans is $V_L/V_R = 0.5$ and 2.0 for the (d) $R \to L$ scans. In the 3rd row, the voltage ratio for (e) $L \to R$ scans is $V_L/V_R = 0.5$ and 2.17 for the (f) $R \to L$ scans. In each row the yellow/red (left column) and the orange/magenta (right column) dwell time histograms are obtained from the left/right pore in $L \to R$ and $R \to L$ directions. Schematics of the electrostatic forces on the DNA in the left/right pore are shown by the blue/green arrows (not to scale). The black envelops represent the exponentially modified Gaussian distribution fit of the dwell time histograms.

[S1] Zhang, Y., Liu, X., Zhao, Y., Yu, J.-K., Reisner, W. & Dunbar, W. B. Single Molecule DNA Resensing Using a Two-Pore Device. Small **14**, 1801890 (2018).

[S2] Liu, X., Zhang, Y., Nagel, R., Reisner, W. & Dunbar, W. B. Controlling DNA Tug-of-War in a Dual Nanopore Device. Small **15**, 1901704 (2019).

[S3] Liu, X., Zimny, P., Zhang, Y., Rana, A., Nagel, R., Reisner, W. & Dunbar, W. B. Flossing DNA in a Dual Nanopore Device. Small **16**, 1905379 (2020).

[S4] Rand, A., Zimny, P., Nagel, R., Telang, C., Mollison, J., Bruns, A., Leff, E., Reisner, W. W., & Dunbar, W. B. Electronic Mapping of a Bacterial Genome with Dual Solid-State Nanopores and Active Single-Molecule Control. ACS Nano, **16**, 5258-5273 (2022).
