# Supplementary material for: Discriminating protein tags on a dsDNA construct using a Dual Nanopore Device
Source: Sci Rep. 2022 Jul 4;12:11305. doi: 10.1038/s41598-022-14609-9 (PMC9253098; doi:10.1038/s41598-022-14609-9)
Supplement: Supplementary file 8 — Supplementary Information 8. [file 41598_2022_14609_MOESM8_ESM.pdf]

Supplementary Material for "Discriminating protein tags on a dsDNA construct using a Dual Nanopore Device"

Swarnadeep Seth[1], Arthur Rand[4], Walter Reisner[2], William B. Dunbar[4], Robert Sladek[3], and Aniket Bhattacharya[1]

[1]*Department of Physics, University of Central Florida, Orlando, Florida 32816-2385, USA*
[2]*Department of Physics, McGill University, 3600 rue university, Montreal, Quebec H3A 2T8, Canada*
[3]*Departments of Medicine & Human Genetics, McGill University, Montreal, H3A 0G1, Canada and*
[4]*Nooma Bio, 250 Natural Bridge Dr, Santa Cruz, CA 95060, USA*

VIII. DWELL TIME DISTRIBUTIONS FOR THE NEUTRAL SIDECHAINS

In our simulation, we have implemented sidechains of different partial charges q_{tag} ranging form $0.0q$ to $0.5q$. The Fig. S1 shows the cumulative dwell time distributions of the seven neutral sidechains for the voltage bias ($V_L/V_R = 0.5$ for the $L \to R$ and $V_L/V_R = 2.17$ for the $R \to L$ scans) matching with the experimental setup. Unlike the charged sidechains the asymmetry almost disappears as the sidechains no longer interact with the extended electric field.

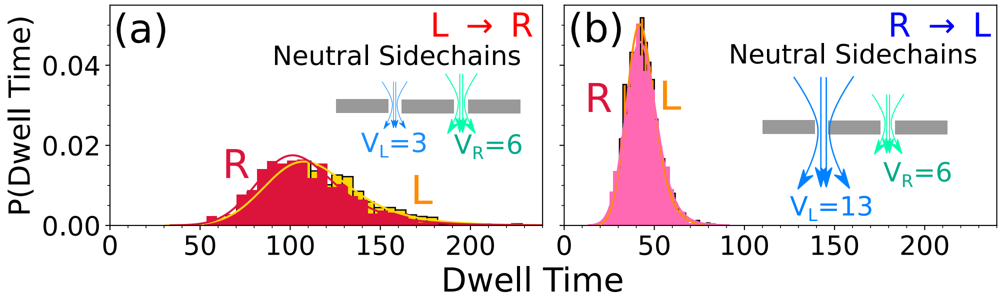

FIG. S1. Cumulative dwell time distributions of the neutral seven tags (sidechains) (a) $L \to R$ and (b) $R \to L$ scans. For the $L \to R$ scans the left pore voltage is $V_L = 3$ and right pore voltage is $V_R = 6$ while for the $R \to L$ translocations $V_L = 13$ and $V_R = 6$. The yellow/red and the orange/magenta dwell time histograms are obtained from the left/right pore in $L \to R$ and $R \to L$ directions respectively. Schematics of the electrostatic forces on the DNA in the left/right pore are shown by the blue/green arrows (not to scale). The black envelops represent the exponentially modified Gaussian distribution fit of the dwell time histograms.
