# Supplementary material for: Discriminating protein tags on a dsDNA construct using a Dual Nanopore Device
Source: Sci Rep. 2022 Jul 4;12:11305. doi: 10.1038/s41598-022-14609-9 (PMC9253098; doi:10.1038/s41598-022-14609-9)
Supplement: Supplementary file 9 — Supplementary Information 9. [file 41598_2022_14609_MOESM9_ESM.pdf]

Supplementary Material for "Discriminating protein tags on a dsDNA construct using a Dual Nanopore Device"

Swarnadeep Seth[1], Arthur Rand[4], Walter Reisner[2], William B. Dunbar[4], Robert Sladek[3], and Aniket Bhattacharya[1]
[1]*Department of Physics, University of Central Florida, Orlando, Florida 32816-2385, USA*
[2]*Department of Physics, McGill University, 3600 rue university, Montreal, Quebec H3A 2T8, Canada*
[3]*Departments of Medicine & Human Genetics, McGill University, Montreal, H3A 0G1, Canada and*
[4]*Nooma Bio, 250 Natural Bridge Dr, Santa Cruz, CA 95060, USA*

IX. TRANSLATING SIMULATION BIAS TO THE EXPERIMENTAL BIAS & PÉCLET NUMBER

In the original experiments [S1–S3] the bias at the left pore is varied from $F\sigma \approx k_BT - 10k_BT$, while the bias at the right pore is larger for $L \to R$ scans. The biases used in the BD simulation should commensurate with these. Please note that in the Fig. 2 of the main article we used a factor of 50 in translating 150 mV, 300mV to 3, and 6 BD simulation units respectively. We will now show the internal consistency by calculating the Péclet number as outlined below.

When the voltage bias is increased from $k_BT \simeq 1$ to 10, the diffusive motion changes over to the drift. Thus, several authors used the Péclet number

$$P_e = \frac{\tau_{\text{relax}}}{\tau_{\text{trans}}} \tag{S1}$$

to compare the applied bias used in the simulation [S4–S6] with those in the experiment. Here, τ_{relax} and τ_{trans} are the relaxation and translocation time for the translocating polymer, thus is a measure of diffusive versus the drift motion. A comparison of Péclet number can provide useful information in this context. Previous studies [S4–S7] were done in reference to a single nanopore. We use a similar argument for the dual nanopore system. However, it is worth pointing out that during flossing the chain does not escape completely, rather a major segment of the chain (90% in our simulation) is scanned back and forth. Therefore, initial conformations of the chain those translocate through the dual nanopore are far from equilibrium and are different from well established studies carried out in the context of a single nanopore where a scaling exponent is sought for the driven translocation for an equilibrated initial chain. The compressed configuration in our study unfolds and translocates faster depending on the degree of compression. Flossing a λ-phage DNA-construct with seven tags in a dual nanopore setup is performed under the voltage bias ΔV_{LR} ranging from $150 - 650$ mV [S2, S3] which results in a typical TOF velocity $v_{TOF}^{expt} \simeq 0.77$ mm/s (Supplementary Material Table S3 of [S3]). Hence, for a 16.6 μm λ-phage dsDNA $\tau_{trans}^{expt} \simeq (16600/0.77)\ \mu s \approx 0.02\ s$.

For $L = 16.6\ \mu$m λ-phage dsDNA used in the dual nanopore experiment [S2] we use the formula by Smith et al. [S8] $D_{bulk}^{expt} = 2.38/L^{0.608} = 0.43\ \mu\text{m}^2/\text{s}$, and the formula for the bulk radius of gyration $(R_g)_{bulk}^{expt} = 0.146L^{3/5}$. This gives $(R_g^2)_{bulk}^{expt} \simeq 0.621\ \mu\text{m}^2$ and $\tau_{relax} = (0.621/0.5)s = 1.24s$. Thus, for the double nanopore experiment Péclet number is $P_e^{expt} = 1.24s/0.02s \approx 60$.

Now, we get the Péclet number for the BD simulation using $\sigma = 16$ nm and the chain's time of flight velocity $\tilde{v}_{TOF} \simeq 0.1$. Please note that the average velocity of the chain will depend on the applied differential bias conditions. The average translocation time from the multiple scans is $\tau_{trans}^{sim} = 1024/0.1 \simeq 10240$. To get the BD simulation relaxation time we use the relation $\tau_{relax} \sim B^2\gamma N^{2.2} = 0.153 \times (1024)^{2.2} = 643057$ in $k_BT/\sigma = 1$ unit [S5] and from there we obtain $P_e^{sim} = 643057/10240 \simeq 63 \approx P_e^{expt} \approx 60$. Thus, this agreement of the Péclet numbers from the experiment and the simulation further justifies and closes the loop why the BD simulation studies capture the essential features of flossing in a dual nanopore device and give further confidence to use this model for analysis of a more complicated mixed system of tags *in silico*.

[S1] Zhang, Y., Liu, X., Zhao, Y., Yu, J.-K., Reisner, W. & Dunbar, W. B. Single Molecule DNA Resensing Using a Two-Pore Device. Small **14**, 1801890 (2018).
[S2] Liu, X., Zhang, Y., Nagel, R., Reisner, W. & Dunbar, W. B. Controlling DNA Tug-of-War in a Dual Nanopore Device. Small **15**, 1901704 (2019).
[S3] Liu, X., Zimny, P., Zhang, Y., Rana, A., Nagel, R., Reisner, W. & Dunbar, W. B. Flossing DNA in a Dual Nanopore Device. Small **16**, 1905379 (2020).
[S4] Saito, T., & Sakaue, T. Process time distribution of driven polymer transport. Physical Review E, **85**, 061803 (2012).

[S5] de Haan, H. W., Sean, D. & Slater, G. W. Using a Peclet Number for the Translocation of DNA through a Nanopore to Tune Coarse-Grained Simulations to Experimental Conditions, Phys. Rev. E **91**, 022601 (2015).

[S6] McMullen, A., de Haan, H. W., Tang, J. X., & Stein, D. Stiff filamentous virus translocations through solid-state nanopores. Nature Communications, **5**, 061803 (2014).

[S7] Storm, A. J., Chen, J. H., Zandbergen, H. W., & Dekker, C., Translocation of double-strand DNA through a silicon oxide nanopore, Phys. Rev. E **71**, 051903 (2005).

[S8] Smith, D. E., Perkins, T. T., & Chu, S., Dynamical scaling of DNA diffusion coefficients, Macromolecules, **29**, 1372 (1996).
